# Association between behavioral patterns and mortality among US adults: National Health and Nutrition Examination Survey, 2007–2014

**DOI:** 10.1371/journal.pone.0264213

**Published:** 2022-02-18

**Authors:** Jiyun Jung, Jeonghwan Lee, Eunjin Bae, Yong Chul Kim, Eun Young Kim, Jangwook Lee, Sung Joon Shin, Yon Su Kim, Jung Pyo Lee, Jae Yoon Park

**Affiliations:** 1 Data Management and Statistics Institute, Dongguk University Ilsan Hospital, Goyang, Korea; 2 Research Center for Chronic Disease and Environmental Medicine, Dongguk University College of Medicine, Goyang, Korea; 3 Department of Internal Medicine, Seoul National University Boramae Medical Centre, Seoul, Korea; 4 Department of Internal Medicine, Gyeongsang National University College of Medicine, Changwon, Korea; 5 Department of Internal Medicine, Seoul National University Hospital, Seoul, Korea; 6 Mental Health Centre, Seoul National University Health Care Centre, Seoul, Korea; 7 Department of Human Systems Medicine, Seoul National University College of Medicine, Seoul, Korea; 8 Department of Internal Medicine, Dongguk University Ilsan Hospital, Goyang, Korea; 9 Department of Internal Medicine, Dongguk University College of Medicine, Goyang, Korea; Ehime University Graduate School of Medicine, JAPAN

## Abstract

Few large-scale studies have been conducted to show the joint effects of mortality associated with physical activity and sedentarism. Therefore, we examined the relationship between all-cause mortality and behavioral patterns among adults in the United States. Data of 17,730 non-institutionalized US civilians aged ≥20 years were extracted from the 2007–2014 National Health and Nutrition Examination Survey. We set the criteria for metabolic equivalents as 600 according to the WHO guideline, and sedentary time as 300 min/day according to the median. The Cox proportional hazards model was adjusted for demographic and lifestyle characteristics. During the 58.54±28.18 months follow-up, all-cause mortality rate was 4% and heart-related and cancer mortality rate was 1%. Participants in the high metabolic equivalents and low sedentary time group had a lower risk of all-cause (hazard ratio = 0.41, 95% confidence interval = 0.34–0.50), cardiovascular (hazard ratio = 0.36; 95% confidence interval = 0.23–0.55), and cancer (hazard ratio = 0.55; 95% confidence interval = 0.37–0.83) mortality, compared to those in the low metabolic equivalents and high sedentary time group. Sufficient physical activity and less sedentary behavior reduce all-cause and cause-specific mortality in adults in the United States, especially cardiovascular mortality among the elderly. Additional nationwide policies to improve behavioral patterns among adults need to be implemented in the United States.

## Introduction

Insufficient physical activity has consistently been reported as a risk factor for major chronic diseases and all-cause mortality in the general population [1]. Additionally, patients with cardiovascular and kidney disease reportedly have higher morbidity and mortality rates when physically inactive [2, 3]. Consequently, the United States (US) Department of Health and Human Services developed the Physical Activity Guidelines for Americans (PAG) in 2008 [4]. However, since then, there has been no increase in the physical activity of adults in the United States (US), in fact, their lifestyle is becoming more sedentary [5].

Sedentary behavior and physical inactivity lead to adverse health outcomes, such as mortality, hospitalization, diabetes mellitus, hypertension, cardiovascular disease, and various types of cancer [6]. Thus, in 2018, the PAG recommended that sedentary time (ST) should be reduced and physical activity increased [7]. However, due to the extensive use of computers, smartphones, televisions, and video game consoles in our society, it is challenging to avoid sedentary behaviors [8]. A few large-scale studies have been conducted to show the joint effects of physical activity and sedentarism on mortality in adults aged over 20 years and older. The black participants aged 40–79 years in the Southern Community Cohort Study (between 2002 and 2009) had 47% greater mortality risk associated with being in the most time sitting and the least active group [9], and similar results were found in a prospective cohort study of 149,077 participants aged over 45 years in Australia during 2006–2009 [10]. Therefore, we examined the relationship between behavioral patterns and death using data from the National Health and Nutrition Examination Survey (NHANES), to examine the association between mortality in the general US adult population and behavioral patterns.

## Materials and methods

### Study population

This study used data of non-institutionalized US civilians who participated in the NHANES. Of the initial 40,617 participants in the NHANES 2007–2014, our analysis included the complete data of 17,730 participants aged ≥20 years on alcohol use, smoking status, diagnosis of chronic disease, income, depression, physical activity, sedentary behavior, education, and body mass index (BMI). Participants were selected using the stratified, multistage probability sampling approach. The NHANES compiled yearly information from interviews, demographics, standardized physical examination, laboratory test, and health-related questionnaire data of approximately 5,000 individuals of all ages to assess their health and nutritional conditions. Health examinations were conducted at mobile examination centers (MEC) in a standardized fashion. We processed NHANES data in four 2-year cycles (2007–2008, 2009–2010, 2011–2012, and 2013–2014) because the questionnaires on physical activity changed in 2007, and mortality data were available only until the 2013–2014 cycle. All participants provided written informed consent, and the National Center for Health Statistics (NCHS) Research Ethics Review Board approved the NHANES protocols (protocol #2005–06 and #2011–17).

### Mortality data

We used a publicly available file from the National Centre for Health Statistics (NCHS) with certified death records from the National Death Index (NDI). We used the information provided by the NCHS regarding the leading causes of death for NHANES 2007–2014 because of the short follow-up times and small sample sizes. Cause-specific deaths were available for heart diseases (I00-I09, I11, I13, and I20–I51) and malignant neoplasms (C00-C97). Follow-up periods were calculated from the date of the interview to the date of death for the deceased or the end of the follow-up period (December 31, 2015) for those who survived.

### Data collection

We collected demographic data such as age, sex, ethnicity, educational level (low: <11^th^ grade, middle: high school graduate/general education curriculum, some college or AA degree, and high: college graduate or above), and family income-to-poverty ratio (FIPR, 0–1·31, >1·31–3·5, and ≥3·5). FIPR was calculated by dividing family income by poverty as reported in the NHANES guidelines, where FIPR >1 correspond to high family income. Five categories of ethnicity were used: Mexican American, other Hispanic, Non-Hispanic White, Non-Hispanic Black, and other. Age was stratified into 20–39, 40–59, and more than 60 years of age. BMI was calculated (from data collected by trained health technicians at MEC) as weight (kg) divided by height in square meter (m^2^). Drinking status was determined from the information obtained at the MEC and was categorized into (1) current drinkers (at least 12 alcoholic drinks in a year), (2) former drinkers (less than 12 alcoholic drinks in a year but drank in the past), and (3) non-drinkers (have never taken alcohol). Smoking status was categorized through a questionnaire into (1) current smokers (smoked at least 100 cigarettes in their lifetime and currently smoke), (2) former smokers (smoked at least 100 cigarettes in the lifetime and are currently non-smokers), and (3) non-smokers (have never smoked). Depression was defined using the Patient Health Questionnaire-9 (PHQ–9) score obtained by the self-reported assessment of nine symptoms with scores of 0 (not at all) to 3 (nearly every day) in the past 2 weeks [11]. Depression severity was divided into mild (score 0–9) and moderate to severe (score 10–27). Hypertension and diabetes assessment were performed using information provided by the participants who were previously diagnosed with these conditions by doctors and other health professionals. Borderline diabetes diagnosis was treated as missing information.

### Behavioral pattern

Physical activity and sedentary behavior were assessed using the Global Physical Activity Questionnaire (GPAQ) developed by the World Health Organization (WHO) [12]. The GPAQ is used to record the intensity, duration, and frequency of physical activity data in three domains (vigorous-and-moderate-intensity activity at work; transport activity; and vigorous and moderate activity during sports, fitness, and recreational activities), as well as the frequency of sitting (inactive periods). Vigorous-and-moderate-intensity activity was defined as physical activity causing significant heart rate increase (lifting heavy loads or construction work for at least 10 min continuously in a typical week) and causing minor heart rate increase (brisk walking or carrying light loads). Transport activity included walking or cycling for at least 10 min in a typical week. When participants responded positively to the three domains of physical activity, the duration (min/week) was measured by multiplying the number of active days (1–7 days) in a week by the mean duration of the activity per day (min/day). Based on the weekly duration for the three domains, we calculated METs scores following the NHANES guidelines describing the ratios of working and resting metabolic rates. Using the MET score for sitting quietly as a baseline, a MET score of four was assigned to moderate work- and leisure-related activity and transport activity, and eight was assigned for vigorous work- and leisure-related activity. The overall METs of the participants were calculated by adding the total time spent doing physical activities during the week multiplied by the matched MET values in all three domains. The WHO recommends at least 150–300 min of moderate-intensity physical activity weekly, 75–150 min of vigorous-intensity physical activity per week, or an equivalent combination of moderate- and vigorous-intensity activity. We followed the WHO recommendation to set the cut-off values for moderate-related physical activity by 150 min, vigorous-related physical activities by 75 min, and METs by 600 on GPAQ guideline [13]. Sedentary behavior was defined as the amount of time (min/day) spent sitting, traveling by car or bus, reading, playing cards, watching television, or using a computer on a typical day before falling asleep [14].

### Statistical analyses

We created Kaplan–Meier curves for all-cause mortality, cardiovascular, and cancer mortality of METs and sedentarism to estimate the crude risk. The various physical activities and sedentary behavior were divided into two categories: moderate activity/moderate work-related activity, moderate leisure-related activity/transportation (<150 and ≥150 min/week, respectively) and vigorous activity/vigorous work-related activity, vigorous leisure-related activity (<75, ≥75 min/week, respectively). In addtion, we set the criteria for METs as 600 according to the WHO guideline and ST as 300 based on the median value of our dataWe used the least active and most sedentary group as the reference group. A Cox proportional hazards model was used to estimate all-cause and cause-specific mortality risks associated with physical activity and sedentary behavior. Data were age-stratified and adjusted by sex, ethnicity, educational level, BMI, FIPR, smoking and drinking habits, hypertension, diabetes, and PHQ-9 score severity. Behavioral patterns were categorized into four groups: MET<600 and ST ≥300 min/day (reference, type 1), MET <600 and ST <300 min/day (type 2), MET ≥600 and ST ≥300 min/day (type 3), MET ≥600, and ST <300 min/day (type 4). We explored the association between mortality and activity patterns in several age groups (20–39, 40–59, <65, and ≥65 years of age). We tested the proportional hazard assumption using the Schoenfeld residuals. All analyses followed the NHANES guidelines considering sample weight, stratification, and clustering in the complex survey design. All the results are presented as hazard ratios (HRs) and 95% confidence intervals (CIs). Data analyses were conducted using the survey package in R statistical software version 4·0·2.

## Results

**Fig 1:**
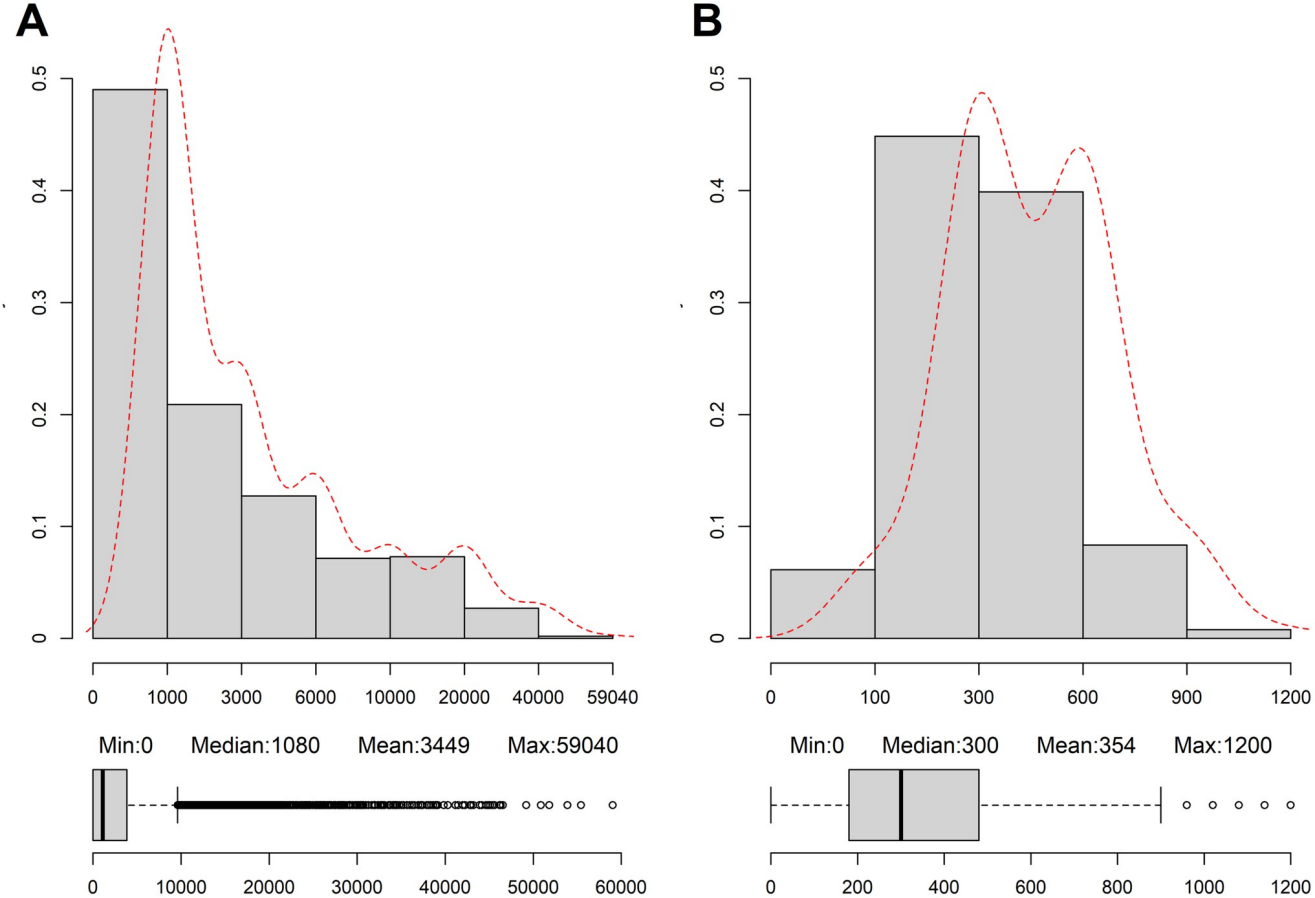
Unweighted distribution of metabolic equivalents (METs) (A) and sedentary behavior (B) in National Health and Nutrition Examination Survey (NHANES) 2007–2014.

Data from the 17,730 adult participants from the initial 40,617 NHANES 2007–2014 data met the inclusion criteria (S1 Fig). The unweighted minimum, median, and maximum MET values were 0, 1080, and 59040, respectively, and 0, 300, and 1200 min/day for ST (Fig 1). When stratified by behavioral patterns, most participants (38%) were in the type 3 category, and people aged 60–80 years comprised 35% of individuals in the type 1 group and 19% of those in the type 4 group (Table 1). More than half of participants had middle education levels, regardless of behavioral patterns. Moreover, compared to the other groups, more participants with FIPR >3.5 belonged the type 3 category. Non-Hispanic White was the predominant ethnicity. Although most participants were overweight or obese, regardless of their behavioral patterns, reported diagnoses of hypertension and diabetes were gradually lower from types 1 to 4. The weighted average and standard deviation of follow-up months for survival, all-cause mortality, cardiovascular disease, and cancer deaths were 59.27 ± 28.09, 41.50 ± 24.66, 41.76 ± 24.83, and 41.23 ± 24.36, respectively.

**Table 1:** Unweighted number (n) and weighted percentages (%) of 2007–2014 NHANES participants (n = 17,730).

	Behavioral pattern				
	Type 1	Type 2	Type 3	Type 4	p-value^a^
n (%)	4679 (25)	2362 (10)	5790 (38)	4899 (27)	<0.001
Age (years)					
20–39	1129 (26)	550 (28)	2438 (43)	2005 (44)	<0.001
40–59	1489 (39)	788 (38)	1906 (38)	1673 (38)	
60–80	2061 (35)	1024 (34)	1446 (20)	1221 (19)	
Sex					
Male	1941 (40)	850 (34)	3165 (54)	2780 (56)	<0.001
Female	2738 (60)	1512 (66)	2625 (46)	2119 (44)	
Race/ethnicity					
Mexican American	462 (6)	570 (14)	519 (5)	951 (11)	<0.001
Other Hispanic	406 (5)	294 (8)	412 (4)	590 (7)	
Non-Hispanic White	2299 (71)	802 (57)	2961 (74)	2152 (67)	
Non-Hispanic Black	1098 (12)	516 (13)	1198 (10)	876 (10)	
Other Race	414 (6)	180 (7)	700 (7)	330 (5)	
Educational level					
Low	1140 (17)	913 (28)	804 (10)	1411 (20)	<0.001
Middle	2511 (55)	1139 (54)	3020 (50)	2638 (58)	
High	1028 (28)	310 (18)	1966 (40)	850 (22)	
Ratio of family income to poverty					
≤1.3	1524 (22)	987 (32)	1557 (18)	1735 (25)	<0.001
>1.3 to 3.5	1732 (36)	928 (40)	1879 (31)	1916 (38)	
>3.5	1423 (42)	447 (28)	2354 (52)	1248 (36)	
Smoking status					
Current	1008 (21)	465 (22)	1179 (18)	1151 (24)	<0.001
Former	1275 (26)	531 (22)	1369 (24)	1107 (24)	
Never	2396 (53)	1366 (57)	3242 (58)	2641 (53)	
Drinking status					
Current	3273 (75)	1466 (68)	4554 (83)	3666 (79)	<0.001
Former	720 (13)	401 (16)	628 (9)	619 (10)	
Never	686 (12)	495 (17)	608 (8)	614 (10)	
BMI (kg/m^2^)					
Underweight (<18.5)	212 (4)	101 (4)	291 (5)	205 (5)	<0.001
Normal (18.5–22.9)	943 (20)	552 (24)	1624 (29)	1358 (29)	
Overweight (23–24.9)	1403 (30)	821 (35)	1891 (35)	1765 (35)	
Obesity (≥25)	2121 (46)	888 (37)	1984 (32)	1571 (31)	
Hypertension					
No	2467 (57)	1398 (64)	4030 (73)	3531 (75)	<0.001
Yes	2212 (43)	964 (36)	1760 (27)	1368 (25)	
Diabetes					
No	3793 (85)	1963 (87)	5283 (93)	4474 (94)	<0.001
Yes	886 (15)	399 (13)	507 (7)	425 (6)	
PHQ-9 score					
Minimal to mild (1–9)	4073 (88)	2064 (89)	5402 (95)	4541 (93)	<0.001
Moderate to severe (10–27)	606 (12)	298 (11)	388 (5)	358 (7)	

Type 1: those who satisfied metabolic equivalents (MET)<600 & sedentary time (ST) ≥300; Type 2: MET<600 & ST <300; Type 3: MET ≥600 & ST ≥300; Type 4: MET ≥600 & ST <300.

NHANES, National Health and Nutrition Examination Survey; BMI: Body mass index; PHQ-9 score: Patient Health Questionnaire-9.

^a^ the significant differences between groups in participants characteristics was determined by using the Pearson chi-squared statistic.

**Fig 2:**
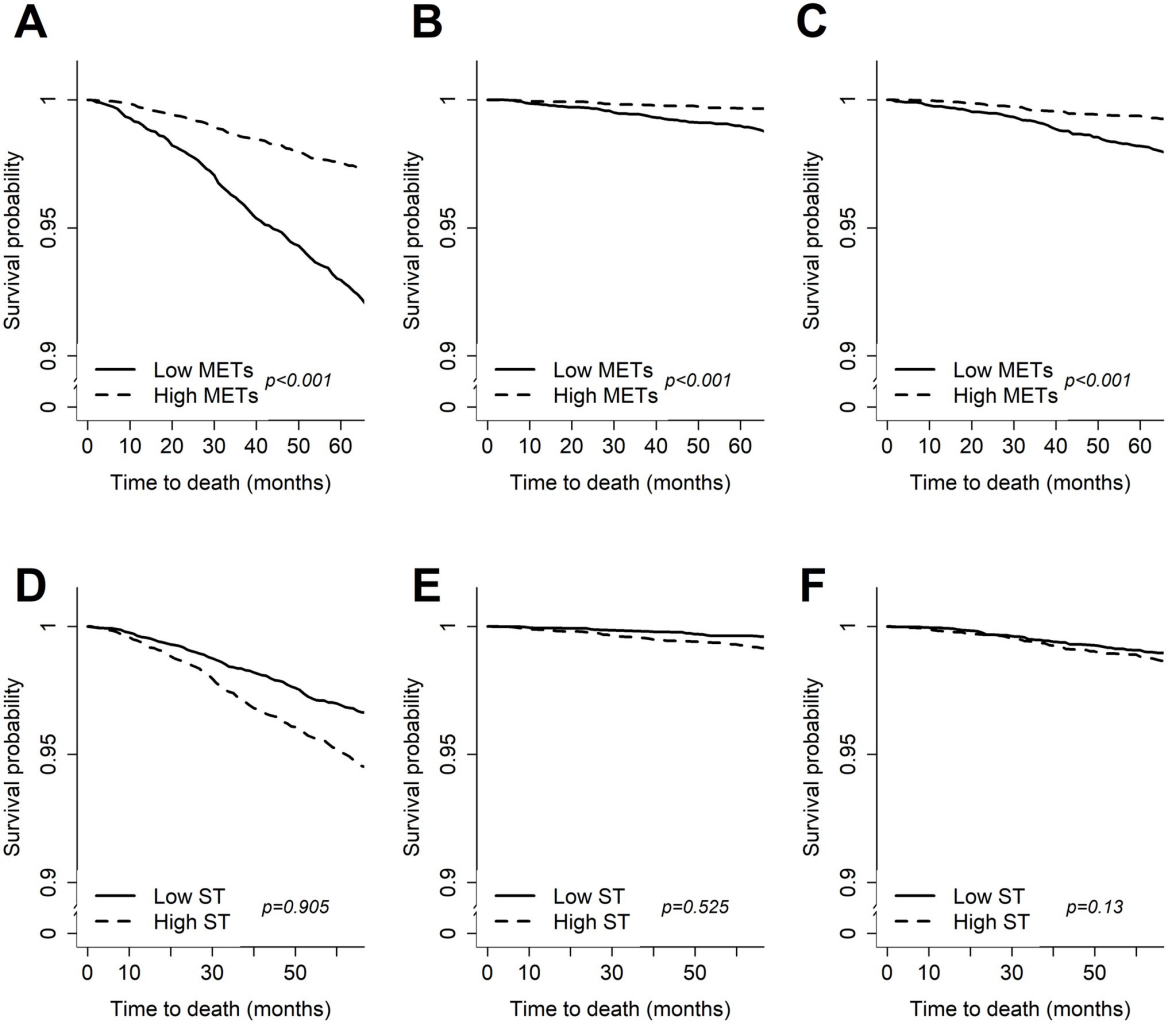
Weighted Kaplan-Meier curve in metabolic equivalents of task score (METs) for: All-cause (A), cardiovascular (B), and cancer (C) mortality and sedentary time (ST) for all-cause (D), cardiovascular (E), and cancer (F) mortality. METs, metabolic equivalents.

Fig 2 shows the weighted Kaplan–Meier curves for the different MET and ST scores. The survival probability of all-cause mortality was significantly lower among physically inactive participants relative to physically active individuals. Physically inactive participants also had a higher mortality risk due to cardiovascular disease and cancer. Furthermore, significant group differences were shown in log-rank test. However, although the probability of all- and cause-specific survival in the high sedentary behavior group was lower than that in low groups, group differences were not significant.

After adjusting for several covariates in the Cox proportional hazards models, we found that physical activity and sedentary behavior affected mortality in the different groups (Table 2). There was no evidence of a violation of Schoenfeld residuals against follow-up time. During the mean follow-up duration (58.54 months), 4% were associated with all-cause mortality and 1% were associated with heart disease and malignant neoplasms. In accordance with the WHO physical activity guidelines, physical activity was associated with decreased risk of all-cause (HR, 0.54; 95% CI, 0.46–0.63), cardiovascular (HR, 0.48; 95% CI, 0.31–0.75), and cancer (HR, 0.60; 95% CI, 0.42–0.85) mortality. Moreover, those who had more vigorous-intensity physical activity (≥75 min/week), moderate-intensity physical activity (≥150 min/week), and sitting time <300 showed 38%, 43%, and 38% lower risks, respectively, of all-cause mortality than those with less vigorous-intensity physical activity (<75 min/week), moderate-intensity physical activity (<150 min/week), and sitting time ≥300 min/week. The HRs of cardiovascular disease mortality were lowest in the vigorous active group (HR, 0.49; 95% CI, 0.26–0.92), followed by the moderate activity group (HR, 0.54; 95% CI, 0.34–0.86). In addition, less sedentary behavior (ST<300 min/day) was associated with decreased death risk due to heart disease (HR, 0.58; 95% CI, 0.42–0.82). In the association between cancer and physical activity, moderate activity (>150 min/week) was associated with a 36% reduction in mortality risk due to malignant neoplasm while vigorous activity (≥75 min/week) did not significantly affect mortality.

**Table 2:** Hazard ratio and 95% confidence interval of all-cause and cause-specific mortality associated with physical activity and sedentary behavior.

	Mortality		
	All-cause	Disease of the heart	Malignant neoplasms
Deaths (n*, %†)	1,073 (4%)	169 (1%)	277 (1%)
METs			
<600	Reference	Reference	Reference
≥600	0.54 (0.46–0.63)	0.48 (0.31–0.75)	0.60 (0.42–0.85)
Vigorous activity			
<75	Reference	Reference	Reference
≥75	0.62 (0.46–0.85)	0.49 (0.26–0.92)	0.72 (0.41–1.25)
Moderate activity			
<150	Reference	Reference	Reference
≥150	0.57 (0.49–0.66)	0.54 (0.34–0.86)	0.64 (0.46–0.89)
ST			
≥300	Reference	Reference	Reference
<300	0.62 (0.54–0.71)	0.58 (0.42–0.82)	0.80 (0.59–1.08)

Stratified by age group and adjusted by sex, race, drinking and smoking status, body mass index (BMI), education, ratio of family income to poverty, hypertension, diabetes, Patient Health Questionnaire-9 (PHQ-9) score; ST: sedentary time; METS, metabolic equivalents;

*unweighted number;

^†^weighted percentage.

**Fig 3:**
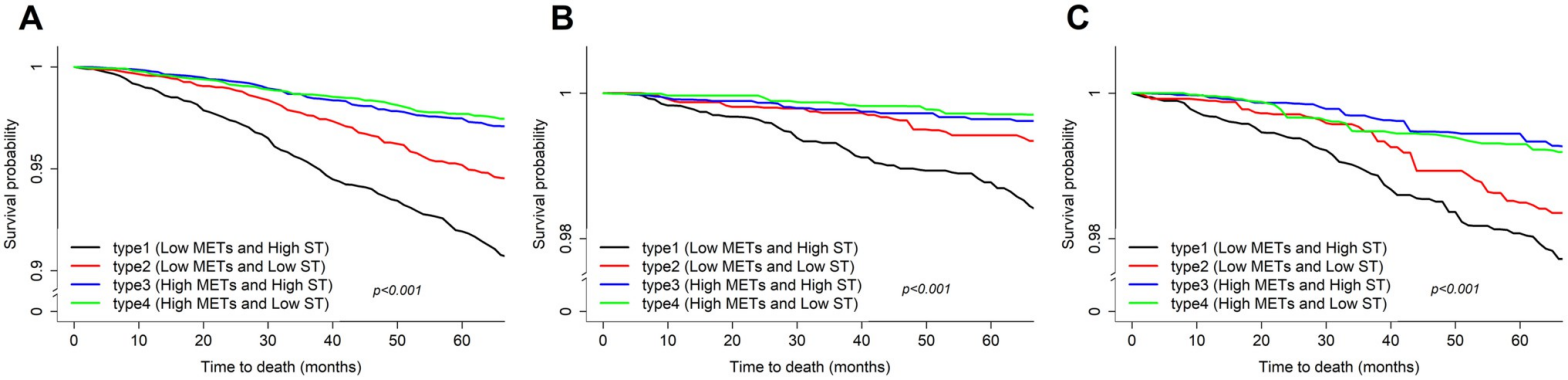
Weighted Kaplan-Meier curve of behavioral pattern (types 1, 2, 3, and 4): For all-cause (A), cardiovascular disease-related (B), and cancer-related (C) mortality. METS, metabolic equivalents; ST, sedentary behavior.

**Fig 4:**
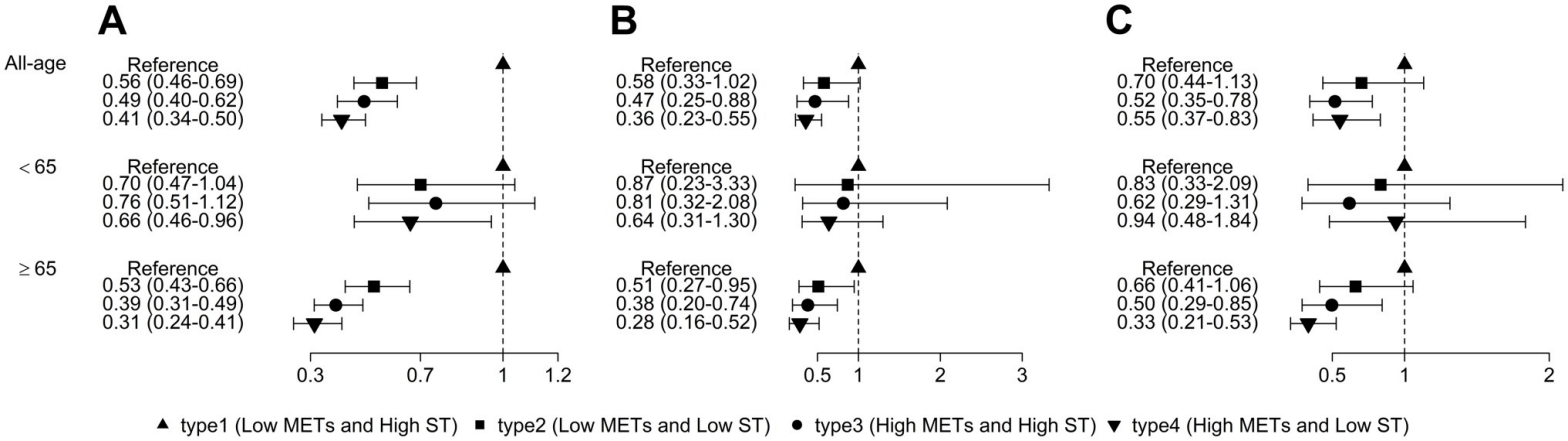
Effects of behavioral pattern on all-cause (A), cardiovascular disease-related (B), and cancer-related (C) mortality stratified by age. METs: metabolic equivalents; ST: sedentary behavior.

When considering behavioral patterns altogether, among all the four behavioral pattern types, the survival probability of those with high ST and low MET were the lowest for all-cause and cause-specific mortality (Fig 3). Fig 4 shows the association between behavioral patterns and death. It shows the joint risk association of MET and ST on the age-stratified mortality causes. Compared to the reference group, those in the type 2 (low MET and low ST) and type 3 (high MET and high ST) groups had a HR of 0.56 (95% CI, 0.46–0.69) and 0.49 (95% CI, 0.40–0.62), respectively. Moreover, those in the type 4 group showed a 59% lower risk of all-cause mortality. Similar results were found for cause-specific mortality. In the subgroup analysis, the HRs for all-cause, cardiovascular disease-related, and cancer-related mortality among participants aged ≥65 years who were more physically active and less sedentary were 0.31 (95% CI, 0.24–0.41), 0.28 (95% CI, 0.16–0.52), and 0.33 (95% CI, 0.21–0.53), respectively. In participants <65 years of age, less sedentary and physically active behavioral patterns were significantly associated with decreased all-cause mortality (HR, 0.66; 95% CI, 0.46–0.96).

## Discussion

Our study clarified the distinct effects of physical activity and sedentary behavior on all-cause, cardiovascular, and malignant tumor mortality in US adults, using the 2007–2014 data of the NHANES. Participants with physically active behavioral patterns had a lower mortality risk than those with a sedentary lifestyle. The joint effects of low ST and high METs were particularly associated with decreased mortality risk among the elderly.

Previous studies results are consistent with the results of our study. In a prospective cohort study of female nurses conducted in the US from 1988–1992, women with consistent MET scores >10.4 h/week had a lower risk of type 2 diabetes than those with MET scores ≤2 h/week (relative risk, 0.59; 95% CI, 0.46–0.75) [15]. Moreover, the risk of hypertension in people with MET scores ≥10 h/week was reduced (relative risk, 0.94; 95% CI, 0.91–0.96) compared to that of a less physically active group (MET score <10 h/week) [16]. The implementation of METs enabled comparison between various physical activity patterns worldwide [17], and similar HR results were found for cardiovascular disease in participants with moderate (MET scores 300–600, HR, 0.88; CI, 0.82–0.84) and high (MET scores ≥600, HR, 0.75; CI, 0.69–0.82) physical activities [18]. Furthermore, increased ST has emerged as a risk factor for all-cause mortality (HR, 1.22; 95% CI, 1.09–1.41), cardiovascular disease (HR, 1.15; 95% CI, 1.10–1.19), cancer (HR, 1.13; 95% CI, 1.05–1.21) [19], high blood pressure in children [20], and breast cancer in African American women [21]. Previous studies also emphasized the risk of a high sedentary behavior, consistent with our results. The highest risks of inflammatory diseases [22], all-cause mortality [23], and obesity [24] have been observed among individuals with inactive and high ST.

We found positive joint effects of higher physcial activity and lower sedentary behavior on mortality compared to those with lower acitivty and higher sitting time. In addtion, our study also showed a lower risk in the type 3 group than that in the type 2 group, indicating that physical activity had a more positive effect on reduced mortality than ST. Continuous aerobic exercise can lead to positive health outcomes through various biological mechanisms. These include increased high-density lipoprotein cholesterol concentration causing changes in lipid-lipoprotein profiles [25], and improved glucose homeostasis and coronary blood flow [26]. Furthermore, physical activity has been shown to improve psychological factors, such as self-efficacy and self-concept, through regulatory changes in the hypothalamic-pituitary-adrenocortical axis, a neuroendocrine system involved in metabolism and stress regulation [27].

Our finding of age-specific association showed that active behavioral patterns strongly reduced mortality risk in the elderly aged over 65 years than the youner age group. As people age, their level of physical activity generally decrease, and a sedentary lifestyle leads to low energy expenditure, increased risk of weight gain, and an adverse metabolic profile [28]. Therefore, the benefits of a healthy behavioral pattern may be greater in older people with less mobility than in younger groups [29]. In addtion, insignificant cause-specific association was found in the younger age group, which may be due to the fewer number of deaths in this group within the relatively short observation period with mortality rates of cardiovascular and cancer diseases of 0.3% (n = 45) and 0.7% (n = 107), respectively.

Physical activity played an important role in lowering the risk of cancer death; however, our findings suggest that moderate-intensity physical activity (<150 min/week) was more beneficial than vigorous physical activity. Our results are consistent with those of a study which explored sporting activity and showed that people who performed exercises had a lower risk of prostate, upper digestive, and stomach cancers, but a higher risk of bladder cancer [30]. Additionally, no significant inverse relationship was observed between moderate or vigorous physical activity and ovarian cancer [31]. This might be because moderate physical activity in cancer survivors mitigates depressive symptoms [32]. We also found that active behavioral patterns were more strongly associated with decreased cardiovascular disease-associated deaths than all-cause and malignant tumor mortality, suggesting that active behavior improves cardiopulmonary function and has heart-protective effects.

This study employed a large and representative sample of the US general population. The information was obtained through regular and standardized questionnaires from the NHANES 2007–2014. The measurement of physical activity was based on the GPAQ, a highly standardized format that allows comparisons between groups and populations worldwide. Our results emphasize the effects of physical activity behavioral patterns on mortality in the US general population, reflecting the sample weight, stratification, and clustering involved in the complex survey design. However, our study had two major limitations. First, respondent interview-derived data on physical activity may have involved recall bias and measurement error, even if questions were asked by trained interviewers. Second, drinking and smoking habits and information on hypertension and diabetes were self-reported and may not have been objectively estimated.

## Conclusions

Physical activity reduces all-cause and cause-specific mortality in the US adults, especially cardiovascular disease-associated mortality in the elderly. Additional nationwide policies to improve health behavioral patterns need to be implemented to improve the life quality of adults in the US.

## Supporting information

S1 Fig Flow chart of participants in the National Health and Nutrition Examination Survey (NHANES) 2007–2014. PHQ-9 score: Patient Health Questionnaire-9; BMI: Body mass index.
(DOCX)
