# Supplementary material for: Association between behavioral patterns and mortality among US adults: National Health and Nutrition Examination Survey, 2007–2014
Source: PLoS One. 2022 Feb 18;17(2):e0264213. doi: 10.1371/journal.pone.0264213 (PMC8856565; doi:10.1371/journal.pone.0264213)
Supplement: S1 Fig — PHQ-9 score: Patient Health Questionnaire-9; BMI: Body mass index. (DOCX) [file pone.0264213.s001.docx]

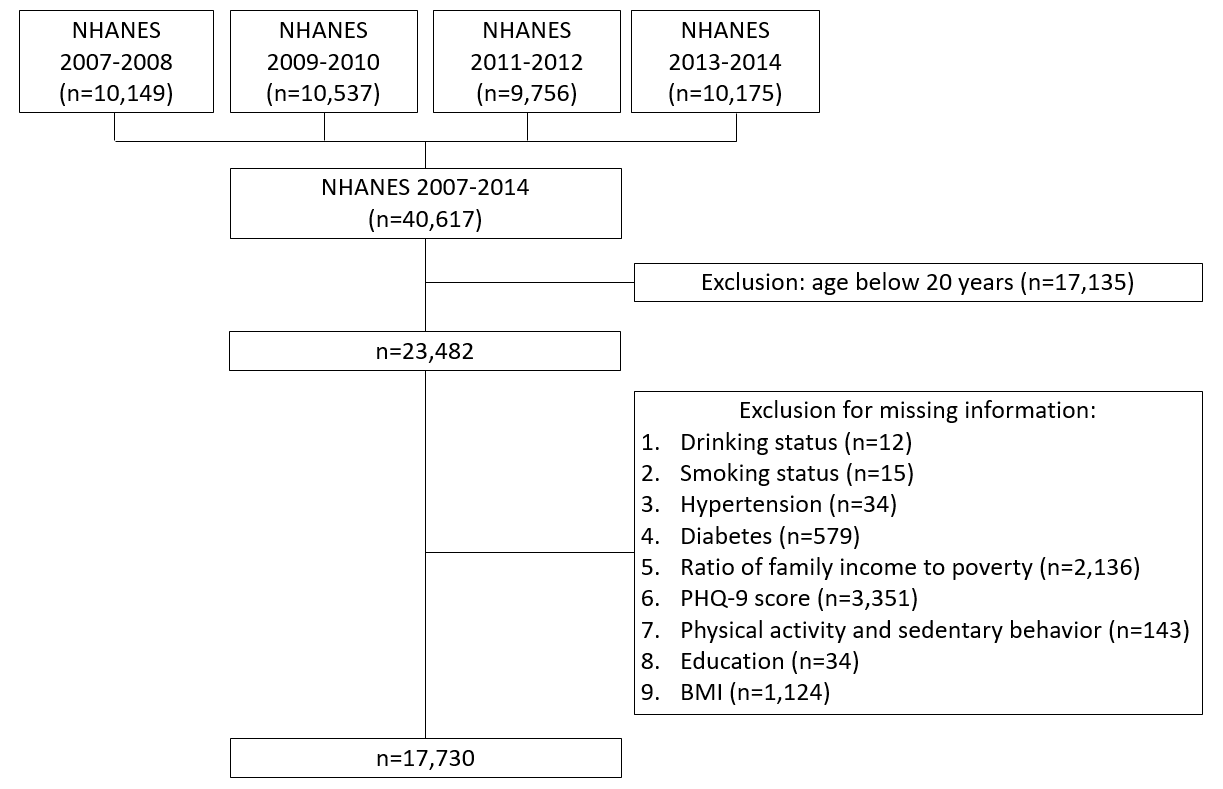


S1 Fig. Flow chart of participants in the National Health and Nutrition Examination Survey (NHANES) 2007-2014

PHQ-9 score: Patient Health Questionnaire-9; BMI: Body mass index
